# Temporal transcriptional profiling of host cells infected by a veterinary alphaherpesvirus using nanopore sequencing

**DOI:** 10.1038/s41598-025-87536-0

**Published:** 2025-01-25

**Authors:** Dóra Tombácz, Zoltán Maróti, Péter Oláh, Ákos Dörmő, Gábor Gulyás, Tibor Kalmár, Zsolt Csabai, Zsolt Boldogkői

**Affiliations:** 1 Department of Medical Biology, Albert Szent-Györgyi Medical School, University of Szeged, Somogyi u. 4, Szeged, 6720 Hungary; 2 MTA-SZTE Lendület GeMiNI Research Group, University of Szeged, Somogyi u. 4, Szeged, 6720 Hungary; 3 Department of Pediatrics, Albert Szent-Györgyi Medical School, University of Szeged, Somogyi u. 4, Szeged, 6720 Hungary; 4 Department of Dermatology, Medical Faculty, University Hospital Duesseldorf, Heinrich- Heine University Duesseldorf, Duesseldorf, Germany

**Keywords:** Cell biology, Microbiology

## Abstract

In our research, we performed temporal transcriptomic profiling of host cells infected with Equid alphaherpesvirus 1 (EHV-1) by utilizing direct cDNA sequencing based on nanopore MinION technology. The sequencing reads were harnessed for transcript quantification at various time points. Viral infection-induced differential gene expression was identified through the edgeR package. The identified genes were segmented into six groups based on their kinetic characteristics. The initial three clusters encompass immediate-early response genes, typically transcription factors and elements of antiviral signaling pathways. These genes were either upregulated (cluster 1) or downregulated (clusters 2 and 3) during the early infection phase. The remaining three clusters include late response genes. In these categories, it is challenging to determine whether changes in gene expression are directly connected to the viral infection or merely side effects of the infection. A study of gene associations using the STRINGDB software revealed several gene networks that might be directly impacted by the virus. We also explored whether gene co-expression could be a result of their collective regulation by upstream transcription factors using the Gene Regulatory Network database. Finally, our differential transcript usage (DTU) analysis identified a number of genes that exhibited altered proportions of transcript isoforms in comparison to non-infected cells. Thus, our analysis revealed that EHV-1 infection not only alters host gene expression but also leads to differential use of transcript isoforms, particularly splice variants.

**Supplementary Information:**

The online version contains supplementary material available at 10.1038/s41598-025-87536-0.

## Introduction

Equid alphaherpesvirus 1 (EHV-1) is a veterinary pathogen affecting horses and belongs to the Varicellovirus genus of alphaherpesviruses^1,2^. EHV-1 infection in horses is associated with myeloencephalopathy, a neurological condition, and its symptoms include upper respiratory tract disease, abortion, neonatal death, and fatal myeloencephalopathy^3^. EHV-1 contains a large, linear, double-stranded DNA genome of approximately 150 kbp in size. The short region of the genome is flanked by inverted repeats^4^, and contains 76 protein-coding genes^5^. Five of these genes (ORF1, 2, 67, 71, and 75) have no homologs in other annotated alphaherpesvirus genomes^6^. EHV-1 enters cells through a receptor-mediated process^7^, and this can occur via endocytosis or fusion between the cell membrane and the viral envelope^8^. Besides productive infection, the virus can also enter a latent state in primary sensory neurons^9^.

Long-read RNA sequencing (LR-RNA-Seq) approaches have been instrumental in analyzing structural and dynamic aspects of transcriptomes. Despite short-read sequencing (SRS) offering high base accuracy and coverage, it falls short in identifying transcription start sites (TSSs), transcription end sites (TESs), splice sites, overlapping transcripts, and polygenic RNA molecules^10^. If SRS is used, a complementary technique such as CAGE-Seq is required for mapping the TSSs^11^. Long-read sequencing (LRS) techniques developed by Oxford Nanopore Technologies (ONT) and Pacific Biosciences (PacBio)^12,13^ can sequence full-length cDNA or native RNA (only ONT) sequences, at the cost of a higher sequencing error rate and lower throughput^14–16^. The ONT approach is especially affected by a high error rate, but this does not pose a significant problem in transcriptome analyses if well-annotated genomes are available. While conserved motifs of gene families create difficulty for SRS in identifying genes and RNA molecules, LR-RNA-Seq does not encounter this issue. Moreover, the ONT technique enables the detection of RNA modifications without requiring base conversion^17–19^.

Several herpesvirus transcriptomes have been studied using different LRS techniques, including the PacBio synthesis-based sequencing platform and the nanopore sequencing from ONT^10^. A number of studies have examined the transcriptomic effects of alphaherpesviruses on host cells during productive infection. For example, Hu and colleagues^20^ examined the effect of Herpes simplex virus type I (HSV-1) on the expression of host cell genes and found that in human primary fibroblasts the type of transcriptomic changes was related to the cellular functions affected by the lytic infection, with SRS revealing that stress response and nuclear transport genes primarily exhibited alternative polyadenylation changes, cell cycle genes showed mostly alternative splicing changes, and neurogenesis-related genes rarely underwent these modifications. Additionally, another study^21^, using long-read RNA sequencing, identified six distinct gene clusters in host cells with significantly altered expression kinetics during bovine alphaherpesvirus type 1 (BoHV-1) infection. Their analysis revealed three functional groups: genes related to basic cell functions, such as morphogenesis, cell cycle regulation, and aerobic respiration, were generally downregulated, while genes involved in antiviral response, transcription, RNA decay, translation, and protein folding were notably upregulated during infection. Transcriptomic profiling of HSV-1 in human primary fibroblasts has identified gene expression changes associated with stress responses, nuclear transport, and alternative splicing^22,23^. Furthermore, transcriptomic profiling of murine cytomegalovirus infection using a dual RNA-seq approach has highlighted host cell responses to viral latency and lytic replication^24^. Similarly, studies on Kaposi’s sarcoma-associated herpesvirus have identified host responses that modulate viral replication during lytic infection^25^. Human cytomegalovirus (HCMV) latency has also been explored at the single-cell level, uncovering elusive viral transcripts and novel regulatory mechanisms^26^. Furthermore, dual transcriptomics has been applied to study interactions between Ostreid herpesvirus 1 and its Pacific oyster host, identifying mechanisms of differential susceptibility across host families^27^.

Previous transcriptomic studies have examined changes in EHV-1 gene expression in peripheral blood mononuclear cells (PBMCs) during viremia, identifying immune-related pathways and differentially expressed genes (DEGs)^28^.

In this work, we utilized a time-course assay to examine the effect of EHV-1 infection on host cell gene expression. This transcriptome analysis was performed using direct cDNA (dcDNA) sequencing, a non-amplification-based technique, on the ONT MinION platform.

## Results

### Differential expression analysis of host genes

In this experiment, we examined changes in the expression of host genes following EHV-1 infection in rabbit kidney (RK-13) epithelial cells over a 48-hour period (mock, 1, 2, 4, 6, 8, 12, 18, 24, and 48 h post-infection) using dcDNA sequencing on the ONT MinION platform. We selected this cell line because it is the most commonly used for propagating EHV-1, primarily due to its ease of handling and ability to produce a high virus yield. Moreover, the rabbit serves as a valuable model organism for studying the molecular pathogenesis of EHV-1.

**Fig. 1:**
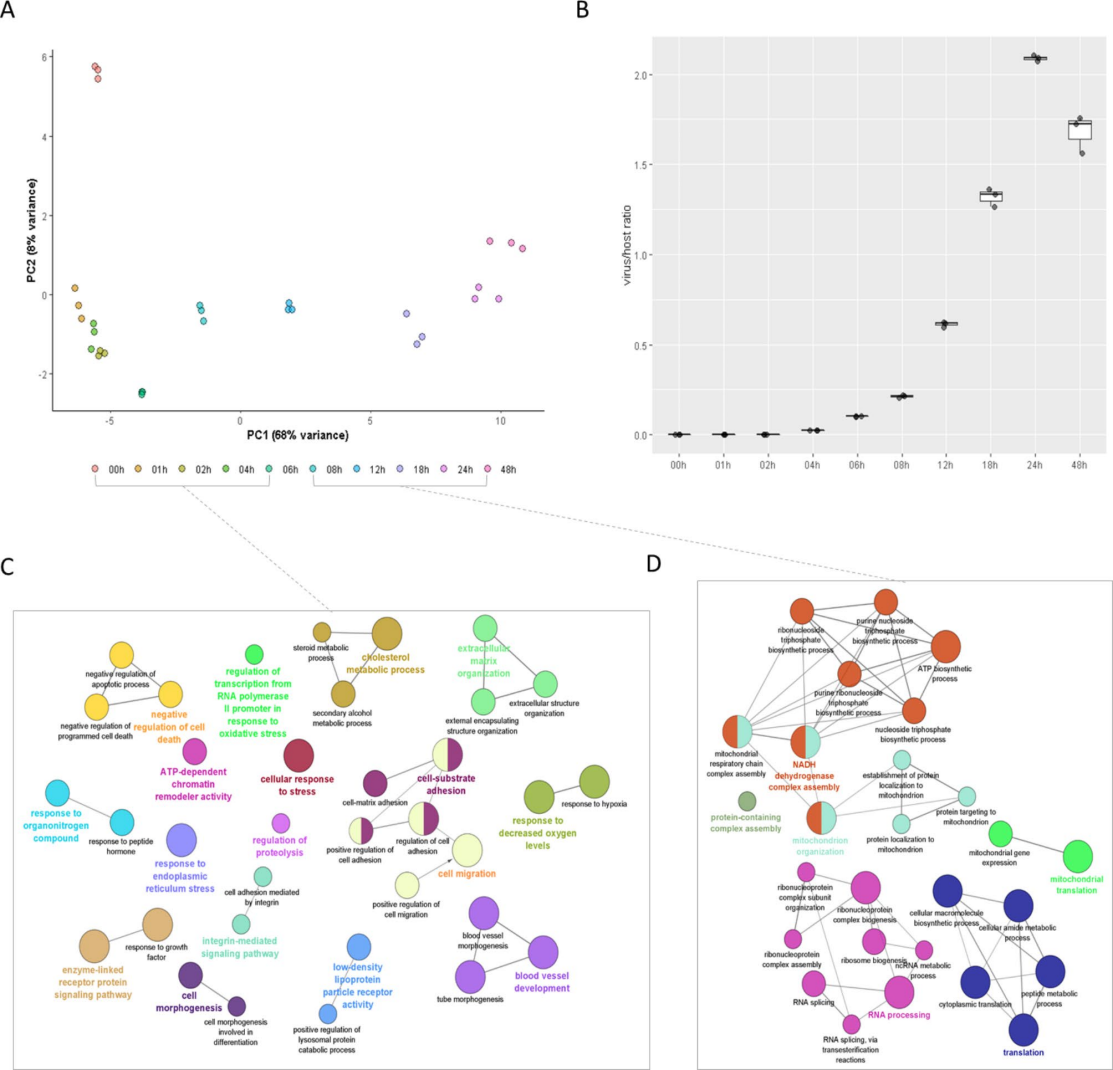
Key host gene expression changes distinguish early vs. late responses to herpesviral infection. A: Principal Component Analysis (PCA) plot of the infection time course. The percentage of gene expression variance explained by PC1 and PC2 is shown in brackets. B: Ratio of sequencing reads originating from the virus and the host. X-axis: infection timepoint, Y-axis: relative ratio (viral reads/host reads). C: Selection of top significantly enriched Gene Ontology biological processes in the E phase of infection, based on differentially expressed genes (log2 fold-change > 1, adjusted p-value < 0.05) from 0 h vs. 2 h to 6 h samples. D: Top Gene Ontology terms from the L phase of infection, comparing gene expression between [2 h to 6 h] vs. [8 h to 48 h] samples. The enriched terms highlight the wide-ranging processes affected during the E phase (including integrin signaling, stress response, and anti-apoptotic signaling), contrasted with the reduced functionality focused on viral translation and virion assembly in the L phase.

From the raw gene counts, we identified 7,308 genes, each having more than ten transcript reads in every one of the three replicates for different time point samples. Principal component analysis (PCA) demonstrates that variability between samples from each timepoint is minimal. Moreover, it highlights the biological patterns across time points through the distinctly segmented distribution of samples between mock (0 h) and infected samples, as well as across the infection time points (along PC2 and PC1, respectively, as shown in Fig. 1A). The relatively high variance in gene expression between the mock and 1-hour samples is followed by much lower variability between the 1- to 6-hour samples. PC1 then illustrates the gradual increase in both qualitative and quantitative changes from early (E) infection to later time points (Fig. 1A). This apparent shift is also consistent with the exponential pattern of viral expansion, as indicated by the relative ratio of host to viral read counts during infection (Fig. 1B). It is notable that by 48 h, a slight rebound is apparent; however, host read counts remain dramatically low and may only reflect minor variation. To explore the hypothesis that the shift in kinetics reflects distinct host regulatory changes between the early (E) and late (L) phases of infection, we first devised a grouped comparison of the ten sampled timepoints, structured to align with the known replication process of EHV-1, as indicated by the PCA ordination. In this high-level analysis, significantly differentially expressed genes (DEGs, with log2 fold-change > 1 and adjusted FDR < 0.01) are compared between mock and all samples from [1 h to 6 h] (the ‘early contrast’) and between samples from [1 h to 6 h] and [8 h to 48 h] (the ‘late contrast’). Notably, in the early contrast we find a wide range of significantly altered pathways by Gene Ontology (GO) term enrichment (Fig. 1C). These outline viral entry (“integrin-mediated signaling”, “LDL receptor activity”) and host defense responses, such as “negative regulation of apoptosis”, stress response and proteolysis, alongside several other processes indicating morphological changes and cell recruitment, cytokine stimuli. Interestingly, the late contrast highlights a narrower, more defined set of processes, dominated by RNA splicing, translation, and protein-containing complex assembly (Fig. 1D). While this is to be expected, our approach helps to single out key molecular players indicating the host cell’s conversion into an effective viral replication machinery. Host gene expression correlated to the virus-host ratio changes has been determined via Spearman correlation (**Supplementary Table ****S1**). Interestingly, the most anti-correlated genes not only include the anti-viral candidate gene SAT1, but several 20 S proteasome subunits, implicated in the antigen-presenting capacity of endothelial cells^29^. Unsurprisingly, the most positively correlated genes include ribosome subunits, indicating a dramatic increase in translation capacity.

## Unsupervised clustering analysis reveals distinct host regulatory patterns in gene expression

**Fig. 2:**
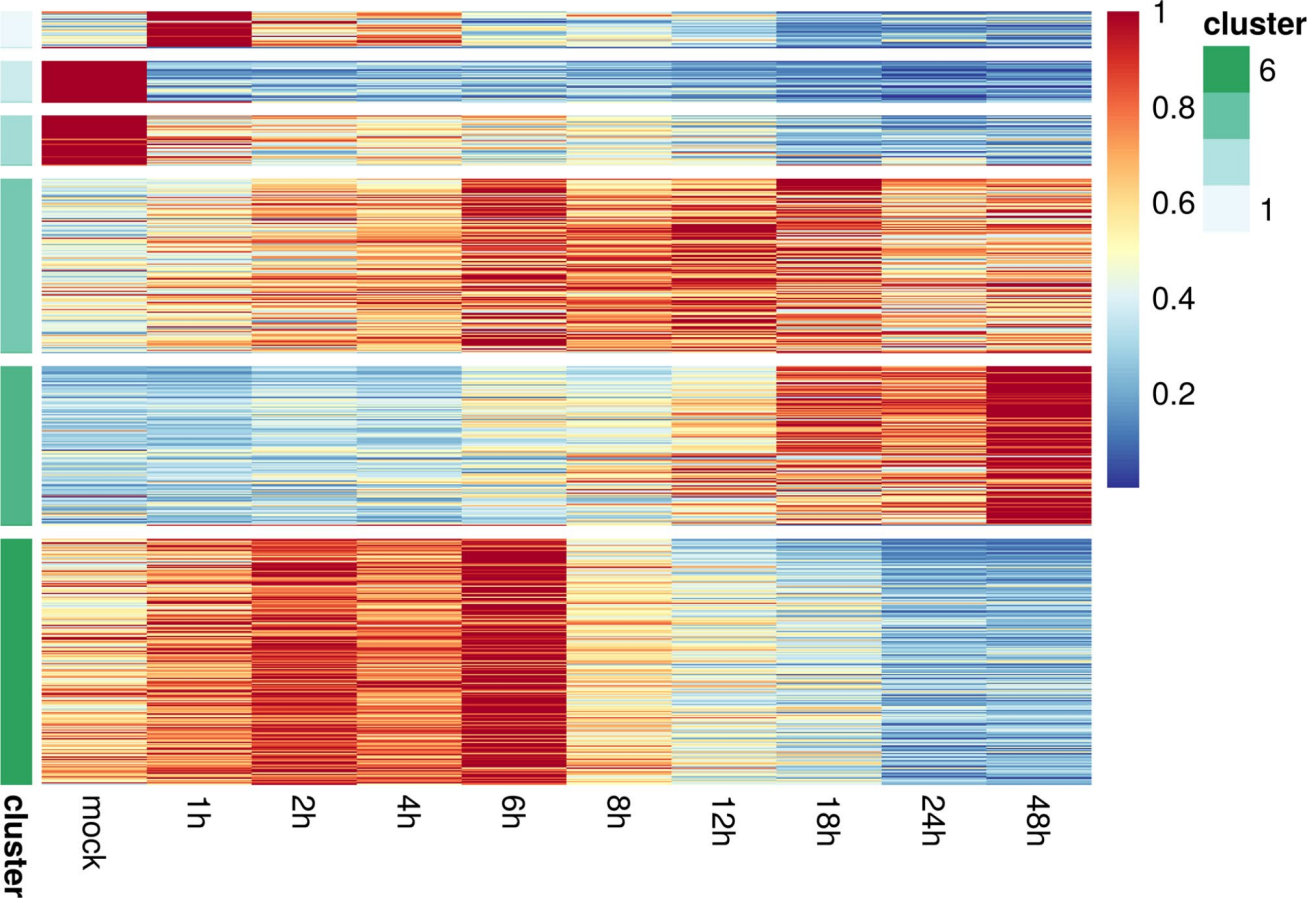
Heatmap of genes with significant differential expression during EHV-1 infection. The relative changes in the host gene expression caused by EHV-1 infection results in gene co-expression clusters that correlate with the temporal dynamics of viral entry and replication.

To complement the coarse-level comparison of E and L phases and more precisely assess the direction of specific changes, a more refined clustering analysis was carried out, identifying distinct gene expression patterns (Fig. 2). By comparing each sampled time point on a one-to-one basis, we identified a refined set of 577 genes that exhibited significant differential expression (FDR < 0.01) between the mock and 1–48 h time points (**Supplementary Table S2**). We then converted the time series of the expression levels of DE genes to a relative scale, portraying the expression changes between the sampling time points, based on their maximum expression level. Subsequently, genes were clustered according to their scaled expression profiles, facilitating the recognition of genes that displayed co-expression (a similar expression pattern throughout the virus infection). This technique surpasses calculations with absolute expression levels of genes as there is a considerable difference between those. Utilizing this method, we were able to categorize six distinct groups of genes that demonstrated modified expression profiles during the course of infection (Fig. 2, **Supplementary Table S3**). In addition, Gene Ontology biological processes and GO molecular function annotations were used for functional characterization of separate clusters. The significant outcomes (FDR < 0.05) of the analysis for each cluster and GO terms are detailed in **Supplementary Table S4**.

## Characterization of immediate-early and early response clusters

**Fig. 3:**
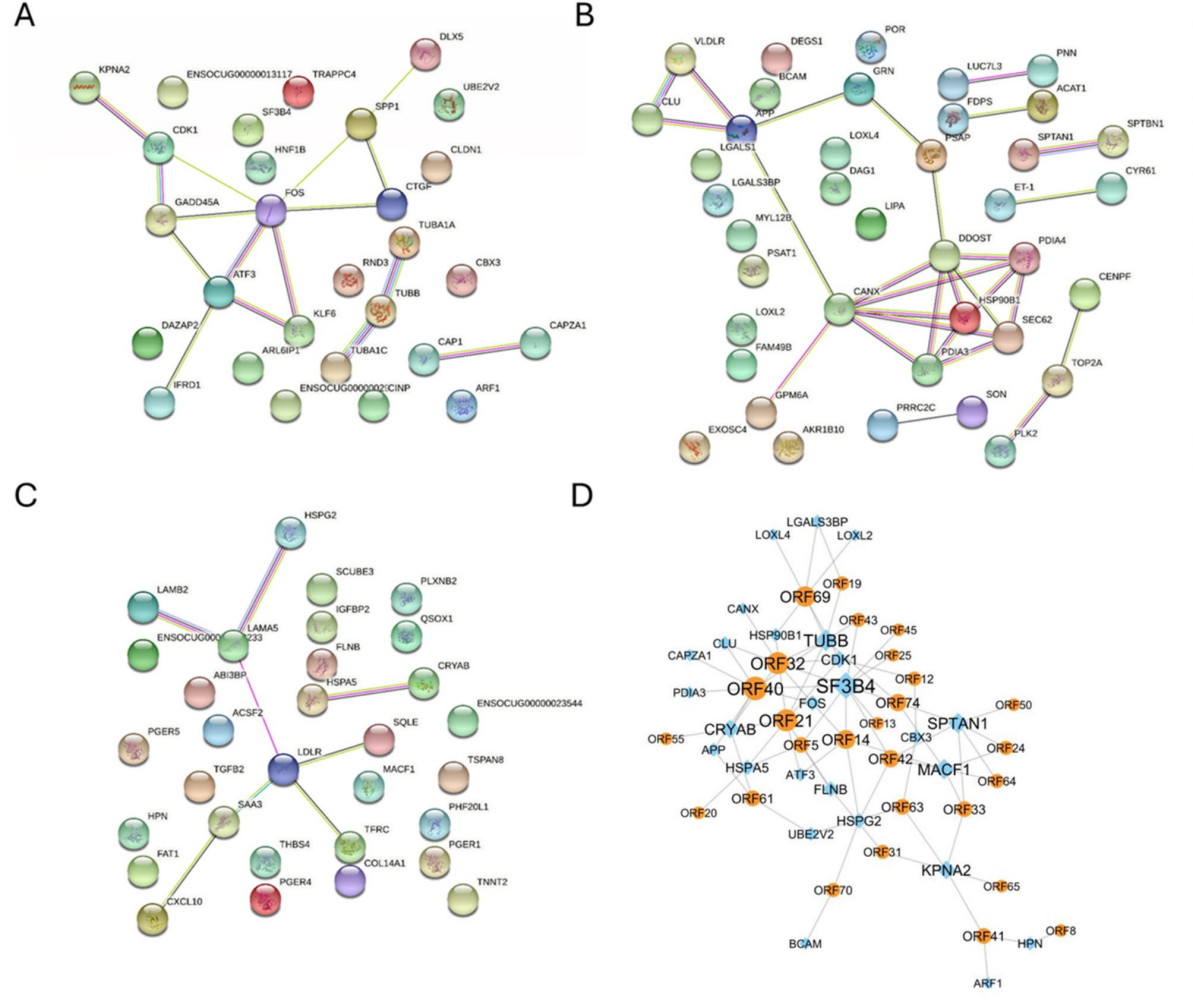
Inferred protein-protein interactions characterizing herpesviral infection. A-C STRING protein and gene interaction networks of gene co-expression clusters no. 1, 2 and 3, respectively. D Predicted protein-protein interaction network between the products of significantly differentially expressed host genes in clusters 1–3 and herpesviral proteins. Orange nodes: EHV-1 proteins, blue nodes: host proteins, connecting edges: significant predicted interaction between nodes.

Cluster 1 was composed of 28 genes that exhibited a significant, transient up-regulation at 1 h, which then significantly decreased at all subsequent time points. This cluster includes genes for transcription factors (ATF3, FOS, and HNF1B), cell-cycle regulators (CDK1), cytokines (SPP1), cell signaling (CTGF, CAP1), tubulin encoding (TUBB), heterochromatin-binding protein (CBX3), stressful growth arrest (GADD45A), translocation through the nuclear pore (KPNA2), cell adhesion (CLDN1), and components of the DNA replication complex (CIMP), among others. Cluster 2 (comprising 33 genes) and Cluster 3 (40 genes) contained genes whose expression was sharply downregulated at 1 h and further reduced at all subsequent time points. This downregulation was more pronounced for Cluster 2. Key inflammatory cytokines and acute-phase mediators (CXCL10, SAA3, THBS4) are down-regulated immediately upon infection, alongside TGFB2, low-density lipoprotein receptors, structural constituents and adhesion factors. Interestingly, no over-represented pathways were detected in cluster 1. However, for cluster 2, we identified an over-representation of genes located in the extracellular matrix structural constituent, structural molecule activity, amyloid-beta binding, heparin binding, integrin binding, protein-containing complex binding, cell adhesion molecule binding, and calcium ion binding GO molecular functions. This cluster also showcased a wide variety of GO biological processes, such as cell adhesion, extracellular matrix organization, multi-organ morphogenesis regulation, among others. In cluster 3, over-representation of genes implicated in protein folding in the endoplasmic reticulum, adenylate cyclase-modulating G protein-coupled receptor signaling pathway, steroid metabolic process, lipid metabolic process, platelet degranulation, secretion by cell, and export from cell GO Biological processes were detected. Furthermore, we explored the potential gene-gene associations within these clusters using STRINGDB^30^. This examination revealed multiple gene networks in each cluster (Fig. 3A-C). Besides the JUN/FOS and ATF3 immune-response related pathways, tubulin and heat shock protein interactions also feature prominently in the networks, suggesting stress response and viral entry-related activation. Importantly, the network analysis affirmed that co-expression was the primary interaction type among the genes of these clusters (**Supplementary Tables S5**,** S6**). To provide further context, building on the predicted virus-host protein interaction network of HSV-1 published in^31^, we extracted the predicted interactions for significant DEGs in the immediate-early (IE) and E clusters (Fig. 3D). Again, the interaction of tubulin, spectrin and several cytoskeletal cross-linking genes, heat shock and related crystallines, alongside immune mediators is demonstrated, with several viral glycoproteins and ribonucleotide reductase. As a reference, we provide rabbit-to-human orthology scores and EHV-1 to HSV-1 protein similarity mapping as supplementary material (**Supplementary Tables S10** and **S11**, respectively).

Finally, to investigate whether the co-expression of genes in clusters 1–3 might be due to shared regulatory elements, we gathered all high-confidence associations between transcription factors and genes from the Gene Regulatory Network database (GRNdb; human embryo E-MTAB-3929, **Supplementary Table S7**). Our analysis discovered numerous transcription factors (TFs) shared among the genes of these clusters. Furthermore, cluster 1, which displayed a transient up-regulation, comprises three transcription factors (ATF3, FOS, and HNF1B). These transcription factors, as potential pivotal regulators, may control a significant number of downstream genes involved in antiviral defense.

## Characterization of early-late and late response clusters

**Cluster 4**, containing 141 genes, demonstrated an increasing expression throughout the course of the infection compared to mock. This cluster correlates moderately with the virus-host ratio, however it is also dominated by ribosomal genes and translation/elongation facilitators. **Cluster 5** comprised 129 genes that had relatively low expression in the mock and showed a later response to viral infection that peaked at 48 h. Accordingly, genes are highly positively correlated with the virus-host ratio and are dominated by mostly ribosomal genes and long non-coding RNAs, complementing cluster 4 and the genes found among “late contrast” DEGs. Even though the expression kinetics differed slightly between clusters 4 and 5, they shared several overlapping pathways, such as ribosomal RNA assembly, mRNA processes, transcription initiation, viral transcription, translation initiation, protein localization, protein transport, and others that account for the complete replication and packaging of the herpesvirus within the host cell. Lastly, **Cluster 6**, with 200 genes, whose expression slightly elevated until 6 h compared to mock, after which they were extensively down-regulated until the end of the infection. Importantly, this large cluster is dominated by cellular respiration, splicing and key life-supporting metabolic pathways, and follows the kinetic shift in viral expansion starting between 6- and 8-hours post-infection. We found pathways related to the host cell’s energy and metabolic functions, responsible for cell energy supply, catabolic processes, and various signaling pathways. To a lesser extent, pathways in cluster 6 that overlapped with some pathways in clusters 4 and 5 (RNA binding, protein transporter activity) were also identified.

## Differential transcript usage analysis

We used IsoQuant to identify and count the host transcripts at different time points for each replicate, as this approach combines both reference-based and *de novo* transcript discovery. Differential transcript usage (DTU) analysis was performed to compare transcript isoforms between mock and infected cells across consecutive time points. This analysis identified 720 genes with distinct transcript isoforms, and sufficient gene/transcript counts in the dataset (see Methods for details on the filtration criteria). Except for one TES variant, all the detected isoforms are splice variants produced through alternative splicing. **Supplementary Table S8** contains the p-values of significant (*p* < 0.05) genes with DTU and the estimated transcript fractions in the mock and at various infection time points. Several genes showed DTU at different time points, suggesting long-term changes. Notably, the mock vs. 1 h time point data showed the highest number of DTU genes (*n* = 19) compared to subsequent time points, where 2–9 genes exhibited DTU. This suggests that some early immediate responses may be attributed to DTU in genes. We identified 36 genes with significant differences in transcript isoform expression across various stages of infection compared to non-infected cells. All these transcripts correspond to splice isoforms. However, in two genes, we observed both TSS and TES variants; in three genes, only TES variants; and in six genes, only TSS variants were detected (see Table 1 for the list of genes and **Supplementary Table S8**, sheet “Transcript isoforms,” for the list of transcript variants). Notably, many of the genes displaying differential transcript usage (DTU) are involved in mRNA processing (e.g., DEK, ZMAT5, HNRNPA3, PPIH, TRA2A, TRA2B, and BUD31). Interestingly, several ribosomal proteins (MRPL42, MRPL55, and RPS24) also exhibited DTU across infection time points (**Supplementary Table S9**). Remarkably, 9 genes (ATG101, DEK, DENR, EEF1E1E, PS15, MRPL42, NSFL1C, RSL1D1, and SDHD) showed DTU exclusively during the first hour post-infection, while 2 genes (CUEDC2 and HAX1) exhibited DTU within the first two hours.

**Table 1:** The list of genes specifies differentially expressed transcript isoforms as a result of EHV-1 infection.

Gene	Variant			Gene	Variant		
	Splice	TSS	TES		Splice	TSS	TES
APP	+			LOC100344938	+		+
ATG101	+			MRPL42	+		
BCL2L1	+	+		MRPL55	+	+	
BUD31	+	+		NSFL1C	+		
CD44	+			PPIH	+	+	
CUEDC2	+			RACGAP1	+		
DEK	+			RPS24	+	+	
DENR	+			RSL1D1	+		
EEF1E1	+		+	SDHD	+		
EPS15	+		+	TFG	+		
ETFA	+			TMEM59	+		
GDI2	+			TMPO	+		
HAX1	+			TPM1	+	+	+
HDAC3	+			TRA2A	+		
HM13	+			TRA2B	+		
HNRNPA3	+	+	+	TRPC4AP	+		
IMMT	+			UQCRQ	+	+	
KTN1	+			ZMAT5	+		

### Comparing EHV-1 infection effects on gene expression in different cell types

To contextualize our findings, we compared the results of our study with a previous investigation of the transcriptomic response to EHV-1 infection in PBMCs^28^. The differences in species (rabbit vs. horse) and cell types (epithelial cells vs. PBMCs), along with the fact that the previous study was conducted in vivo while ours was performed in vitro, led to distinct patterns of gene expression and regulatory pathways. **Supplementary Table S10** summarizes the key differences and similarities between the two studies, emphasizing the cell-type-specific nature of the host response to EHV-1 infection. For instance, CXCL10 is strongly upregulated in PBMCs during infection but significantly downregulated in epithelial cells, reflecting the distinct roles these cell types play in immune response and viral dissemination. Moreover, ribosomal activation and stress response pathways dominate in epithelial cells, whereas immune-related pathways, such as interferon signaling, are more pronounced in PBMCs. These differences highlight the diverse mechanisms employed by the host to combat EHV-1 and underscore the importance of studying multiple cell types to gain a comprehensive understanding of host-pathogen interactions.

## Discussion

In this research, we employed an LR-RNA-Seq method (ONT MinION, dcDNA-Seq) to explore the host cell’s response to EHV-1 infection over a span of 48 h. We utilized annotated sequencing reads to compute the abundance of transcripts at the specified time points. First, we inspected the major differences in gene expression between the E and L stages of infection by forming two coarse contrasts, with a separation point at 6 h. Based on the virus/host mRNA ratio, the 6 h timepoint represents the start of exponential viral replication, which is also evidenced by the distribution of samples derived from principal component analysis (PCA) based on the top 1,000 most variable host genes. We then further categorized the identified genes into six clusters according to their scaled expression profiles. Each cluster includes genes demonstrating similar expression patterns throughout the entire period of viral infection we examined. We note that in the later stage of infection, it is challenging to discern if the changes in gene expressions are functional or if they simply signify transcriptional noise in cells undergoing disintegration. The most intriguing clusters are clusters 1–3, which contain genes that are either transiently upregulated (cluster 1) or downregulated (clusters 2 and 3) in the very early stages of infection. It is important to acknowledge that the limitations inherent in our experimental design may lead to the inclusion of genes within these clusters that exhibit altered expression, potentially as a consequence of the physical stimuli introduced by the applied infection protocol.

Cluster 1 contains the transcription factors ATF3, FOS, and HNF1B, which may govern downstream genes involved in antiviral defense. The ATF/CREB transcription factor family, comprising nearly twenty members, embodies a collection of leucine zipper proteins^32^. ATF3 is a marker for cellular stress and regenerative response and has been seen to be induced upon infection by the Japanese encephalitis virus, regulating cellular antiviral pathways in the absence of type I interferons^33^. The c-fos nuclear phosphoprotein, encoded by the FOS gene, forms a complex with the JUN/AP-1 transcription factor. This IE gene is initially expressed in response to various external stimuli, such as cytokines, growth factors, hormones, and stress^34^, playing a crucial role in controlling cell proliferation, differentiation, and survival, as well as immune responses and more. The HNF1B gene has a broad range of functions, including suppressing the innate immune response^35^. The exact role of this gene in defending against the virus remains unclear. On the other hand, marked upregulation of IE genes is observed in secreted phosphoprotein 1 (osteopontin, SPP1), which strongly correlates with interferon gamma (IFNG) expression in a variety of inflammatory conditions and infections^36–38^. However, during the course of infection, these usually well-correlated Th1 cytokines show alternating patterns in IE upregulation of SPP1 contrasted by the L upregulation of IFNG (**Supplementary Figure ****S1**).

The inflammatory cytokine CXCL10 is found in cluster 2 and is the only CXC family cytokine expressed in our model system. It is instrumental to initiating host response through its receptor CXCR3 and has been implicated in a variety of viral infections^39,40^. Interestingly, here we find CXCL10 among the top down-regulated genes at the first hour of infection, suggesting the silencing of immune cell recruitment during early response, and the expression stays similarly suppressed throughout the time-course (**Supplementary Figure ****S1**).

TGFB2 shows IE downregulation in cluster 2, thus the widespread and context-dependent effects of TGFB signaling^41^ appear to be suppressed upon infection. Importantly, the inhibition of TGFB signaling affected not only the lytic infection efficacy, but also the latency period of HSV-1 in mouse models^42^ CTGF, a main target of TGFB signaling also displays steady downregulation following a small peak in expression at 2 h. Two of the main SMAD pathway elements, SMAD2 and SMAD4 are less robustly regulated during infection, with SMAD2 showing minor upregulation at L timepoints (**Supplementary Figure ****S1**).

Another representative of the IE downregulation pattern is the low-density lipoprotein receptor (LDLR) gene. While LDLR has been known as a receptor for some RNA viruses^43^, it has recently been shown that LDLR expression effectively attenuates Gammaherpesviral infection in a cell-type specific manner^44^, while it is downregulated in infected cells, a pattern closely replicated in our study as well (**Supplementary Figure ****S1**). In addition, the very low-density lipoprotein receptor (VLDLR) is known to serve as an evolutionarily conserved binding site for the E1 and E2 viral glycoproteins of a range of alphaherpesviruses^45^, and shows a linearly decreasing trend during infection (**Supplementary Figure ****S1**). Interestingly, a further key inflammatory player that shows dramatic and lasting IE dowregulation is the apolipoprotein Serum amyloid A3 (SAA3), an acute phase reactant long known to be highly induced upon infection^46^ and tested as a candidate marker of equine alphaherpesvirus infection^47^. However, as our results show, this gene is also effectively silenced in infected cells (**Supplementary Figure ****S1**). Integrins are well known as herpesvirus receptors^48^, and we observe the IE downregulation especially in ITGAV, known to bind the gH glycoprotein^49^, but also ITGAX and ITGA3.

Late clusters, on the other hand, show the overrepresentation of ribosomal complexes alongside protein synthesis and assembly, while genes anti-correlated with the virus-host ratio represent declining mitochondrial function and putative anti-viral agents. One such agent, spermidine-spermine N1-acetyltransferase (SAT1), has been shown to deplete polyamines, thereby effectively reducing the replication of RNA viruses^50,51^; however, they are less well characterized in Herpesviridae^52^.

Using our LR-RNA-Seq data, we investigated isoform-specific changes in rabbit cells through DTU analysis, where the genome annotation is less detailed compared to that of humans. Interestingly, our approach, which combines reference-guided and *de novo* transcript discovery, indicated that differential transcript usage is most prominent at the early infection time points (**Supplementary Table S8**,** S9**). This contrasts with DEGs, which increase in number and fold regulation in the later phases of infection. The majority of genes exhibiting DTU are involved in epigenetic and transcriptional regulation (HDAC3, DEK, ZMAT5, and RSL1D1), RNA processing (TRA2A, TRA2B, BUD31, HNRNPA3, and PPIH), autophagy (ATG101 and TMAM59), apoptosis (BCL2L1, HAX1, and TMAM59), energy production (ETFA, IMMT, SDHD, and UQCRQ), as well as cytoskeletal (RACGAP1 and TPM1) and vesicular functions (EPS15, TFG, and NSFL1C). This suggests the virus may hijack the host’s transcriptional machinery, starting as early as the initial phases of infection. We note that splicing is rare in alphaherpesvirus transcripts, with the ICP27 protein playing a key role in blocking unnecessary mRNA splicing that would otherwise lead to dysfunctional viral mRNAs^53^. High GC-content host genes, which account for ~ 1% of the host transcriptom^54^, have also been shown to be targeted by ICP27. Many of the key genes exhibiting DTU as a result of EHV-1 infection have been described as targets of other herpesviruses. For example, BCL2L1 (Bcl-xL) is upregulated by Epstein-Barr virus (EBV)^55^ and HCMV^56^ to prevent apoptosis, while HDAC3 is targeted by EBV^57^ and HCMV^58^ to regulate gene silencing and viral latency.

Taken together, our high-resolution time-course analysis of host response to EHV-1 infection offers a detailed resource on the significant differential gene expression, co-expressed gene clusters and inferred host and viral protein interactions that may provide future targets and intervention opportunities in herpesvirus research.

## Materials and methods

### Cells and viruses

In this study, we used equid alphaherpesvirus 1 (EHV-1) strain MdBio (EHV-1-MdBio)^59^. EHV-1 was propagated in confluent rabbit kidney (RK-13) epithelial cell line (ECACC: 00021715), which is conventionally used for cultivating this virus. Another reason for using RK-13 cells to propagate EHV-1 is that rabbits serve as model organisms for studying the infection mechanisms of this virus^60^. RK-13 cells were cultivated in DMEM (Sigma), supplemented with 10% fetal calf serum (Gibco) and 80 µg of gentamycin per ml (Gibco) at 37 °C in the presence of 5% CO_2_. Virus stock solution was prepared by infecting the freshly prepared cells with 0.1 multiplicity of infection [MOI = plaque-forming units (pfu)/cell]. Infected cells were harvested when complete cytopathic effect was observed. As a next step, three successive cycles of freezing and thawing of infected cells were performed to release of viruses from the infected cells. For the time-course sequencing experiments, the cells were infected with MOI = 4 of EHV-1-MdBio in three technical replicates. For the synchronization of infections, cells were incubated for 1 h at 4 °C, followed by the removal of the virus suspension and washing the cells with phosphate-buffered saline. As a next step, fresh culture medium was added to the infected cells, which were incubated for 1, 2, 4, 6, 8, 12, 18 24 and 48 h post-infection. The term ‘mock’ (0 h) refers to cells incubated for 1 h at 4 °C under the same conditions as the infection experiments, but without the addition of viruses. The time-series dataset was reported in our previous publication^51^. After the incubation, the culture medium was removed, and the infected cells were frozen at -80 °C until further use.

### RNA isolation

The cells underwent RNA extraction following the spin column-based procedure detailed in the NucleoSpin RNA kit from Macherey-Nagel. Initially, the cells were bathed in a lysis buffer with chaotropic ions, a step which served to neutralize RNases. This was followed by the adhesion of DNA and RNA molecules to the silica membrane. All samples were subsequently treated with DNase I to eradicate any lingering genomic (g)DNA. The total RNA was then drawn out into nuclease-free water. Any potentially persistent gDNA was removed with the TURBO DNA-free™ Kit from Invitrogen. The concentration of the samples was ascertained using the Qubit 4.0 fluorometer and Qubit Broad Range RNA Assay Kit, both from Invitrogen. The Agilent TapeStation 4150 was employed for quality control, and only those samples with RIN scores of 9.2 or higher were selected for cDNA production for subsequent procedures.

### Purification of polyadenylated RNA

The poly(A)^+^ RNA fraction was isolated from the total RNA samples using the Oligotex mRNA Mini Kit from Qiagen. Briefly, the samples’ volumes were adjusted to 250 µL using RNase-free water. The samples were then treated with 15 µL of Oligotex suspension and 250 µL of OBB buffer, both supplied in the Qiagen kit. The samples were subsequently heated to 70 °C for a duration of 3 min, before being cooled to 25 °C for 10 min. Following this, the mixtures were spun down at 14,000×g for 2 min, after which the supernatants were disposed of. A quantity of 400 µL of OW2 wash buffer, also from the Oligotex kit, was added to the samples before they were transferred onto the spin columns from the Qiagen kit. They were then spun at 14,000×g for 1 min. This wash step was repeated once more. Ultimately, the polyadenylated RNA fraction was collected from the membrane by introducing 50 µl of heated elution buffer (EB, Qiagen kit). The RNA was collected in 60 µl EB, and a secondary elution step was also performed to ensure maximum yield.

### ONT MinION – dcDNA sequencing

- Both poly(A)+-enriched and poly(A)+-enriched samples processed with Terminator were employed to construct libraries for direct cDNA sequencing on the ONT MinION device. The ONT dcDNA Sequencing Kit (SQK-DCS109) was used according to the instructions provided in the kit’s manual. Briefly, the RNA samples were combined with the VN primer (VNP; from the ONT kit) and 10 mM dNTPs, then heated to 65 °C for 5 min. Following this, 5x RT Buffer, RNaseOUT (from Thermo Fisher Scientific), and Strand-Switching Primer (SSP; from the ONT Kit) were incorporated, with the mixtures then heated to 42 °C for 2 min. The first cDNA strand was produced by adding Maxima H Minus Reverse Transcriptase enzyme (from Thermo Fisher Scientific) to the samples. RT and strand-switching reactions took place at 42 °C for 90 min, before the enzyme was heat inactivated at 85 °C for 5 min. The RNase Cocktail Enzyme Mix (from Thermo Fisher Scientific) was used to remove RNA from the RNA-cDNA hybrids, with this step carried out at 37 °C for 10 min.
- For the synthesis of the second strand, LongAmp Taq Master Mix [from New England Biolabs (NEB)] and PR2 Primer (PR2P) were utilized. The specific details of the PCR reactions can be found in the provided reference^51^. The fragmented DNAs underwent end-repair and dA-tailing using the NEBNext End repair /dA-tailing Module (NEB) at 20 °C for 5 min and then 65 °C for 5 min. This was succeeded by the ligation of the sequencing adapter at room temperature for 10 min, with the NEB Blunt /TA Ligase Master Mix (NEB) used for this process. The ONT dcDNA libraries were marked using barcodes as detailed in the provided reference^51^ and using the ONT Native Barcoding (12) Kit following the manufacturer’s instructions. The prepared and tethered cDNA libraries (200 fmol/flow cell) were purified and introduced into ONT R9.4.1 SpotON Flow Cells. In total, five flow cells were used for the dcDNA sequencing.
- In order to circumvent possible “barcode hopping,” the earlier time point samples were sequenced separately from those taken at later time points. AMPure XP Beads were utilized after each additional enzymatic process. The samples were then eluted in UltraPure™ nuclease-free water (from Invitrogen), and their concentration was determined with a Qubit 4.0 fluorometer, in conjunction with the Qubit dsDNA HS Assay kit.

### Pre-processing and data analysis

The MinION data underwent base calling using the Guppy base caller version 3.4.1, with the --qscore_filtering feature enabled. Reads achieving a Q-score of more than 7 were mapped onto a combined host genome, which comprised the Oryctolagus cuniculus (rabbit) GCF_000003625.3 and Equid alphaherpesvirus 1 (EHV-1) NC_001491.2 reference genomes. This was accomplished using the minimap2 software, with the “-ax splice -Y -C5” options. We chose to exclude MAPQ = 0, secondary, and supplementary alignments from all subsequent analysis. Reads aligned to the host genome were associated with host genes based on the GCF_000003625.3_OryCun2.0_genomic.gff genome coordinates. We only used reads that corresponded with the exon structure of the host reference genes (employing a +/- 5 bp window for matching exon start and end positions) for the computation of raw gene counts. A summary of mapped reads, virus/host ratio, read length/quality for each time point and experiment can be found in **Supplementary Table S10**.

### Differential expression analysis of host genes

EdgeR_3.24.3 29 along with R version 3.5.1 was utilized for the differential expression (DE) analysis. We excluded host genes from the analysis that had fewer than ten reads in any of the three replicates across all time points, retaining 7,308 genes above the chosen threshold (**Supplementary Table S11**). The replicates were then categorized in EdgeR (utilizing the DGEList function) according to their respective time points (mock, 1 h, 2 h, 4 h, 6 h, 8 h, 12 h, 18 h, 24 h, 48 h). We carried out data normalization (with the calcNormFactors function) using the method="TMM” option, and the robust = True option in the subsequent analysis (with the estimateDisp, glmQLFit, and glmQLFTest functions). The normalized pseudo-counts of DE genes, used for clustering and visualization demonstrated superb reproducibility across the three replicates (an average *R* = 0.994 in all cross correlations between the three replicates and various time points), as reinforced by the first two PCA coordinates. Principal components were calculated exclusively from host genes by selecting the top 1,000 genes with the highest variance, using the R function *prcomp*. The first two principal components were then used for a preliminary DEG analysis, comparing mean gene expression between E (mock vs. 1–6 h time points) and L (mean values of 1–6 h vs. 8–48 h samples) phases. Functional enrichment of the resulting DEG lists was performed in Cytoscape using the ClueGO plugin (Reactome Pathway database), with significant hits identified at an adjusted p-value < 0.05, and the output was manually refined for clarity. To identify genes with significantly altered expression levels across the entire time series, we applied the *decideTests* function, setting a false discovery rate (FDR) threshold of 0.01 and adjusting p-values using the Benjamini & Hochberg procedure (via the *p.adjust* function with the method set to “BH”^52^).

Furthermore, Spearman correlation between normalized gene counts and the virus-host ratio across time points was determined by the corr.test function of the “psych” R package, and significantly correlated genes collected in **Supplementary Table ****S1**.

### Differential transcript usage analysis of host genes

The rabbit reference genome does not have gene annotations of the same high quality as the human datasets. As a result, many tools designed for well-annotated genomes fail to function with the available annotations for our host organism. To address this, we chose the IsoQuant long-read aware analysis tool^61^ for isoform discovery, as it is compatible with both annotated and unannotated genomes. IsoQuant can leverage existing annotation data while also discovering novel transcripts from long-read sequence information that are not present in the gene annotation database

It is important to note that, unlike short-read sequencing, where reads are aligned to the genome and transcripts are modeled based on the distribution of reads, long-read transcript isoforms are identified directly from the sequence data. Since not all reads capture the full transcript (due to fragmentation during wet-lab procedures), a significant number of reads may lack the sequence information needed to differentiate isoforms by their unique exon structure. These partial reads are considered ambiguous for each gene. While such reads can be used in differential gene expression analysis, they are excluded from DTU analysis. Consequently, our data is less suited for DTU analysis. Therefore, although long-read data provides better specificity for identifying different transcript variants, the sensitivity is somewhat lower, requiring greater sequencing depth compared to gene expression analysis

For the IsoQuant analysis, we used the combined GCF_000003625.3 and equid alphaherpesvirus 1 (EHV-1) NC_001491.2 reference genomes, along with the CF_000003625.3_OryCun2.0_genomic.Gtf annotation file, provided with the ‘--genedb’ flag. Each time point and its replicates were analyzed separately. IsoQuant outputs both the observed transcript variants present in the gene annotation file and additional novel genes and transcripts in the standard GTF file format. Unlike short-read DTU analysis tools, which calculate raw and normalized read counts from gene exonic regions, IsoQuant directly outputs the raw and normalized counts of observed transcript variants based on alignment data. Consequently, downstream DTU analysis must be performed with algorithms designed for transcript-level data rather than exon-level counts. We used the DRIMSeq R package^62^62 for DTU analysis

The model consisted of three replicates for each time point. We applied the parameters ‘min_samps_gene_expr = 7, min_samps_feature_expr = 3, min_gene_expr = 10, min_feature_expr = 10’ in the dmFilter() function to filter 720 genes that had at least 10 counts, with data in at least 7 different time points, and at least 3 counts in the transcripts of the gene at a minimum of 3 time points. In our model, we tested for DTU between the mock and expression data at each time point to identify genes with significantly (*p* < 0.05) altered transcript usage during the course of viral infection compared to the mock data.

### Examination of host genes exhibiting differential expression during viral infection

To avoid clustering caused by significant differences in absolute gene expression levels, we normalized the data by dividing each value by the highest expression level observed throughout the viral infection. We executed cluster analysis utilizing the amap_0.8–16 R package Kmeans function with the Euclidean distance method to pinpoint genes with similar expression kinetics during viral infection. This resulted in the identification of six clusters of genes with unique expression profiles (**Supplementary Table S3**). We visualized the gene expression heatmap of these clusters using the pheatmap R package (version 1.0.12). The median of the relative gene expressions for each time point within each of the identified gene clusters was visualized with the ggplot2 R package (version 2.3.3.2) using the geom_smooth function.

Employing the identified subset of genes, we carried out an over-representation analysis for each cluster using PANTHER (version 16.0, using the 2020-12-01 data set release; 10.1093/nar/gkaa1106) software tool. The reference was the 7308 genes that were above the selected expression threshold. We compiled the results of the over-representation analysis (FDR < 0.05) using the Gene Ontology (GO) biological processes and GO molecular functions annotation datasets (**Supplementary Table S4**).

We carried out a STRINGDB network analysis (version 11.5) to uncover possible gene networks in clusters 1–3, where the over-representation analysis revealed little or no enrichment in known pathways. Within these specific gene clusters, we also pinpointed the shared regulatory elements of our DE genes based on the Gene Regulatory Network database (GRNdb) data^63^. For this analysis, we used the Human Embryo (E-MTAB-3929) transcription factor (TF) database, which consists of 1529 single-cell experiments from samples across five different embryonic stages, resulting in 170256 TF-gene associations between 18355 target genes and 695 TFs. We only included the high-confidence TF-gene pairs (annotated in the original SCENIC cisTarget database or inferred by orthology^64^ in our investigation.

Furthermore, orthology mapping between human and rabbit genomes was conducted using the Ensembl comparative genomics database (accessed: 17.08.2024) (**Supplementary Table S12**). Identity percentages of coding sequences and human orthology scores were used. Key receptors, cell structural components, and innate immunity genes were validated as highly conserved between species. For the prediction of herpesvirus-endothelial cell interactions at the protein level (Fig. 3D), the HSV-1 protein-protein network published by Lian et al.^31^ was used. HSV-1 to EHV-1 protein mapping was performed using BLASTp on each pairwise combination of proteins, with significant hits provided in **Supplementary Table S13**. The resulting network was visualized in Cytoscape.

## Electronic supplementary material

Below is the link to the electronic supplementary material.

Supplementary Material 1

## Data Availability

The sequencing datasets generated in this study are available at the European Nucleotide Archive under the accession: PRJEB52190. Novel transcript isoform annotation files generated in this study are deposited and publicly available at Zenodo (doi: 10.5281/zenodo.13893550).
